# The TH1902 Docetaxel Peptide-Drug Conjugate Inhibits Xenografts Growth of Human SORT1-Positive Ovarian and Triple-Negative Breast Cancer Stem-like Cells

**DOI:** 10.3390/pharmaceutics14091910

**Published:** 2022-09-09

**Authors:** Michel Demeule, Cyndia Charfi, Jean-Christophe Currie, Alain Zgheib, Bogdan Alexandru Danalache, Richard Béliveau, Christian Marsolais, Borhane Annabi

**Affiliations:** 1 Theratechnologies Inc., Montréal, QC H3A 1T8, Canada; 2 Laboratoire d’Oncologie Moléculaire, Département de Chimie, Université du Québec à Montréal, Montréal, QC H3C 3P8, Canada

**Keywords:** breast cancer, cancer stem cells, docetaxel, multidrug resistance, ovarian cancer, peptide-drug conjugate, sortilin, TH1902

## Abstract

**Background:** Breast and ovarian cancer stem cells (CSC) can contribute to the invasive and chemoresistance phenotype of tumors. TH1902, a newly developed sortilin (SORT1)-targeted peptide-docetaxel conjugate is currently in phase-1 clinical trial. Whether TH1902 impacts the chemoresistance phenotype of human triple-negative breast CSC (hTNBCSC) and ovarian CSC (hOvCSC) is unknown. **Methods and Results:** Immunophenotyping of hTNBCSC and hOvCSC was performed by flow cytometry and confirmed the expression of SORT1, and of CSC markers CD133, NANOG, and SOX2. Western blotting demonstrated the expression of the drug efflux pumps from the P-gp family members, ABCB1 and ABCB5. The cellular uptake of the fluorescent Alexa^488^-peptide from TH1902 was inhibited upon siRNA-mediated repression of *SORT1* or upon competition with SORT1 ligands. In contrast to docetaxel, TH1902 inhibited in vitro migration, induced cell apoptosis and lead to G2/M cell cycle arrest of the hTNBCSC. These events were unaffected by the presence of the P-gp inhibitors cyclosporine A or PSC-833. In vivo, using immunosuppressed nude mice xenografts, TH1902 significantly inhibited the growth of hTNBCSC and hOvCSC xenografts (~80% vs. ~35% for docetaxel) when administered weekly as intravenous bolus for three cycles at 15 mg/kg, a dose equivalent to the maximal tolerated dose of docetaxel. Therapeutic efficacy was further observed when carboplatin was combined to TH1902. **Conclusions:** Overall, TH1902 exerts a superior anticancer activity than the unconjugated docetaxel, in part, by circumventing the CSC drug resistance phenotype that could potentially reduce cancer recurrence attributable to CSC.

## 1. Background

Ovarian and breast cancers are, among the gynecological cancers, classically treated by surgical excision of the tumor, accompanied by (neo) adjuvant radiotherapy and/or taxanes-based chemotherapy [1,2,3]. Epidemiologic studies further highlight a positive association between obesity and the incidence of many of these gynecological cancers [4,5]. Docetaxel is a taxane that is widely used as a chemotherapeutic agent [6], and when administered by infusion crosses cell membranes through a non-specific passive diffusion process [7]. Docetaxel then promotes cell cycle arrest by inhibiting microtubule depolymerization and inducing apoptosis in rapidly dividing cells [8]. However, serious side effects are often associated with docetaxel use, most notably febrile neutropenia that can lead to serious infections. Toxicity is considered to be the main dose limitation in docetaxel treatment [9,10].

The development of resistance against adjuvant chemotherapies is one of the greatest hurdles to successful cancer treatment, and much of this resistance phenotype has been recently attributed to the presence of cancer stem cells (CSC) residing within the tumors [11,12]. CSC are a subpopulation of slow-growing cells within the tumor mass that are self-renewing, undifferentiated and which can regenerate tumors from which they were derived [13]. The overexpression of multi-drug resistance (MDR) proteins such as those from the permeability glycoprotein (P-gp) members ATP-binding cassette sub-family B member 1 (ABCB1) and 5 (ABCB5) within CSC is one of the major mechanisms by which these cells can survive chemotherapeutic treatment [14]. Consequently, a broad variety of unrelated pharmacological agents, including docetaxel, are prevented from reaching a concentration at which they would be effective by being rapidly extruded from the cell by those membrane efflux pumps [15,16].

Sortilin (SORT1) is a receptor protein which cycles from the cell surface through intracellular membrane bodies [17]. It binds several circulating proteins and peptides, including progranulin and neurotensin, prior to their rapid intracellular internalization [18]. It is also involved in their intracellular trafficking in endosomal vesicles from the plasma membrane to lysosomes through the endocytic pathway [18]. Deregulation in SORT1 functions has been implicated in cardiovascular disease, Alzheimer’s disease, and diabetes [19], whereas its upregulation has been documented in several forms of cancer [20,21,22,23]. Recently, TH1902, a peptide-drug conjugate (PDC) to which two docetaxel molecules were linked to TH19P01 peptide, was generated [24,25,26]. TH1902 was demonstrated to recognize and exploit SORT1 functions, and to efficiently inhibit in vitro cell proliferation, and in vivo growth of xenografts from gynecological cancers including triple-negative breast cancer (TNBC)-derived MDA-MB-231 cells, ovarian cancer-derived ES2 and SKOV3 cells, as well as endometrial cancer-derived AN3-CA cells [24,25]. Interestingly, TH1902 was found to reduce in vitro vasculogenic mimicry (VM) processes in breast and ovarian cancer cells [26]. While some molecular aspects revealing the inter-relationship existence between CSC and VM have been addressed in breast cancer [27], gastrointestinal cancer [28], and melanoma [29], the specific targeting of the CSC subpopulation by TH1902 remains unexplored.

Significant progress was made in reducing cancer recurrence, and the significance of CSC to the growth of tumors and resistance to chemotherapy is well recognized [30]. Strategies are currently on-going to find new treatments which can target CSC-specific markers or signaling pathways by the virtue of nanoparticles [31], nanoparticles with cytotoxic payloads [32], or through targeting epigenetics [33] as well as mitochondrial metabolism [34]. Previous work has shown that TH1902 was able to lead to long-lasting complete tumor growth inhibition in TNBC and ovarian xenograft tumor models, suggesting that it may possibly be also active against the respective CSC subpopulation residing within these tumors [35,36]. This study provides the first evidence for TH1902 targeting of human breast and ovarian cancer stem-like cells, both in vitro and in vivo, whereas unconjugated docetaxel has little impact on these highly chemotherapy resistant cell lines. Furthermore, this study also documents the combined therapeutic efficacy of TH1902 with carboplatin.

## 2. Materials and Methods

*Cell cultures and cell line immunophenotyping.* Human triple-negative breast cancer stem cells (hTNBCSC) and human ovarian cancer stem cells (hOvCSC) were purchased from Celprogen (San Pedro, CA, USA). Cells were grown as monolayers at 37 °C in a humidified atmosphere (5% CO_2_) according to the manufacturer’s instructions using the corresponding expansion and undifferentiation media, as well as matrix pre-coated flasks (Celprogen). SORT1 and CD133 immunophenotyping was performed *in-house* for both cell lines. Briefly, cells were suspended in flow buffer (PBS containing 2% fetal bovine serum) and incubated with anti-CD133 or anti-SORT1 for 45 min at 4 °C in the dark. Samples were then rinsed twice in flow buffer and analyzed using a C6 Accuri flow cytometer (BD Biosciences, San Jose, CA, USA). To detect nonspecific signals, concentration- and isotype-matched nonspecific antibodies were used. SOX2 and NANOG immunophenotyping data were provided from the manufacturer (https://celprogen.com/ (accessed on 1 June 2022)).

*Western blot analysis.* Western blotting was performed as previously described using protein lysates isolated from samples [24]. Membranes were washed in TBST and incubated overnight with primary antibodies against ABCB1 (1/100, Enzo, #ALX-801-002-C100), ABCB5 (1/1000, #140667, Abcam, Toronto, ON, Canada) or SORT1 (1/1000, #612100, BD Biosciences, San Jose, CA, USA) diluted in TBST containing 3% BSA and 0.05% NaN_3_. Membranes were washed in TBST and incubated for 1 h at room temperature with horseradish peroxidase-conjugated anti-mouse or anti-rabbit IgG (1/5000 dilution, Jackson Immunoresearch, West Grove, PA, USA) in TBST containing 5% non-fat dry milk. Membranes were washed again in TBST and signals were detected using enhanced chemiluminescence (Amersham Biosciences, Baie d’Urfé, QC, Canada).

*Uptake assays of fluorescent Alexa^488^-TH19P01.* The cellular uptake assay of Alexa^488^-TH19P01 was performed on undifferentiating matrix pre-coated 12-well plates (Celprogen) as previously described [24]. In some experiments, cells were washed with Hank’s Balanced Salt Solution (HBSS, phenol-free) and incubated for 2 h in HBSS in the presence or absence of 200 nM Alexa^488^-TH19P01 for hOvCSC or 500 nM Alexa^488^-TH19P01 for hTNBCSC, along with (or lacking) pre-incubation with excess unlabeled 50 µM TH19P01 for 15 min. In another experiment, cells were first transiently transfected with 50–100 nM of either a scrambled siRNA sequence (AllStar Negative Control siRNA) or siSORT1 (Hs_SORT_5 FlexiTube siRNA; Qiagen, Valencia, CA, USA) for 24–48 h with lipofectamine 2000 (Thermo Fisher Scientific, Austin, TX, USA). The transfected cells were washed with HBSS then incubated at 37 °C in the presence or absence of 200 nM (hOvCSC) or 500 nM (hTNBCSC) Alexa^488^-TH19P01 in HBSS. For all types of experiments, after 2 h of incubation with the fluorescent peptide, cells were washed with HBSS, trypsinized, washed again and fluorescence was measured in the FL1 channel using a C6 Accuri flow cytometer (BD Biosciences).

*Wound healing assay.* The scratch (wound-healing) assay [37] was used for an initial examination of TH1902’s effects on the hTNBCSC and hOvCSC migration. Cells were plated in 6-well plates (2.8 × 10^5^ cells/well) where they grew for 24 h, following which a scratch was made across the slide using a sterile p200 pipette tip. Cells were washed with serum-free medium to remove the detached cells, then treated for 2 h with serum-free medium containing either vehicle (DMSO), 2 µM docetaxel or 1 µM TH1902 (equimolar amounts of docetaxel). The incubation media were then replaced by rinsing with complete media and cells were next incubated in fresh complete medium. Images were acquired at 0 and 24 h after scratching, with an inverted microscope. The images were analyzed using ImageJ software along with the plug-in to estimate the sizes of the scratched area in each image.

*Cell apoptosis assay.* Annexin V/propidium iodide (PI) staining was performed using an Apoptosis Detection Kit following the manufacturer’s instructions. Briefly, cells were seeded for 24 h onto undifferentiating matrix pre-coated 12-well plates (Celprogen), then incubated at 37 °C for 2 h in a serum-free media in the presence or absence of 2 µM TH1902 or 4 µM docetaxel (equimolar amounts of docetaxel) followed by a subsequent 24, 48, or 72 h incubation in complete growth media. Pre-incubation with 10 μM cyclosporin A or PSC-833 was performed for 30 min prior to treatment. At various points during this treatment, cells were harvested and resuspended at a density of 10^6^ cells/mL in a staining solution of 100 µL of 1X binding buffer containing 5 µL of Annexin V-FITC and 5 µL of PI. Cells were incubated in the dark for 15 min at room temperature before analysis by flow cytometry. The extent of apoptotic cells was measured using the BD Accuri C6 software.

*Fluorescent microscopy.* Cells were grown to 80% confluence onto undifferentiating matrix pre-coated 18 mm microscope cover slips (Celprogen), rinsed with PBS and exposed to serum-free undifferentiating culture medium containing either vehicle (DMSO), 4 µM docetaxel or 2 µM TH1902 at 37 °C. Following incubation for 2 h, the cells were rinsed with complete culture medium and incubated in fresh complete medium for up to 48 h. Cover slips were washed with PHEM buffer (60 mM PIPES, 25 mM HEPES, 10 mM EGTA and 2 mM MgCl_2_, pH 6.9) and fixed for 15 min with 4% paraformaldehyde, then permeabilized with 1% Triton X-100 in PHEM buffer for 5 min and washed again with PHEM buffer. The cells were blocked for 1 h in PBS (2.6 mM KCl, 1.4 mM KH_2_PO_4_, 136.9 mM NaCl and 6.5 mM Na_2_HPO_4_·7H_2_O; pH 7.2) containing 10% normal goat serum and 0.05% Triton and then incubated for 1 h with 1/2000 anti-α-Tubulin primary monoclonal antibody (clone B-5-1-2, Sigma-Aldrich, Oakville, ON, Canada) diluted in washing buffer (PBS containing 5% normal goat serum and 0.025% Triton). Cells were washed with washing buffer and incubated for 1 h with Alexa Fluor^488^-conjugated goat anti-mouse secondary antibody (1/1000; Invitrogen, Carlsbad. CA, USA; #A-11001), washed with diluted blocking buffer, stained with DAPI (2 µg/mL in PBS, Invitrogen; #D1306) for 3 min, washed again in washing blocking buffer and mounted onto slides using Prolong Gold antifade reagent (Invitrogen, P36934). Cell images were digitalized by confocal microscopy (Nikon A1) and analyzed using NIH ImageJ Version 1.4.21 software. The excitation and emission wavelengths used for Alexa Fluor^488^ were 488 nm and 525 nm, respectively. The excitation and emission wavelengths used for DAPI were 404 nm and 450 nm, respectively.

*Cell cycle analysis.* Cell cycling was assessed in serum-deprived media upon cell treatment with vehicle, 4 µM docetaxel or 2 µM TH1902 (equimolar amount of docetaxel) for 2 h, washed once, and further incubated with complete media for 22 h. Some of the treatments also had a 30-min pre-incubation with 10 µM cyclosporin A or PSC-833 to inhibit MDR proteins, followed by a 2-h treatment with either Docetaxel or TH1902, then incubated for 48 h. Cells were then detached with trypsin, centrifuged, and washed twice with PBS. One million cells per sample were then resuspended in 0.3 mL of PBS, added to ice- cold 70% ethanol and incubated overnight at 4 °C. Cells were pelleted, washed, and resuspended in FxCycle™ PI/RNase Staining Solution (ThermoFisher, cat. F10797) for 30 min at 37 °C, and analyzed for their DNA content in the FL2 channel using a BD Accuri C6 flow cytometer.

*In vivoassessment of docetaxel and TH1902 anti-tumor efficacy in combination or not with carboplatin.* Immunosuppressed nude mice can host human xenograft tumor models but triple immunosuppressed models (e.g., NCG mice) are recommended for CSC implantations. Tumor xenografts were therefore established by subcutaneous inoculation of only 10^3^ hTNBCSC or hOvCSC, resuspended in 100 µL of HBSS-Matrigel (#356231, Corning; 50:50), into the right flank of young adult, female, homozygous NCG mice (NOD-*Prkdc^em26Cd52^Il2rg^em26Cd22^*/NjuCrl (Charles River Laboratories, Laval, QC, Canada) under light isoflurane anesthesia. Three days following implantation, mice received weekly treatments of paclitaxel, docetaxel, TH1902 or vehicle by intravenous (IV) tail vein injections with carboplatin alone or in combination via intraperitoneal (IP) injections at the doses indicated in the figure legends. Injectable solutions were prepared as follows: TH1902 was solubilized at 10 mg/mL in 10% Tween-80 in D5W (pH 4.3, *w*/*v*), then further diluted with D5W. Docetaxel was solubilized at 25 mg/mL in ethanol and Tween-80 (50/50, *v*/*v*) then further diluted with D5W. Paclitaxel was solubilized at 25 mg/mL in ethanol and Cremophor EL (50/50, *v*/*v*) then further diluted in saline; carboplatin was solubilized at 50 mg/mL in water then further diluted in a solution of 30% PEG400 and 5% Tween-80 in D5W (*w*/*v*); vehicle composition and preparation mimicked the highest dose of TH1902 preparation. Tumor growth was measured using two-dimensional measurements taken with an electronic caliper, and tumor volume was calculated according to the following formula: tumor volume (mm^3^) = π/6 × length × width^2^. Animal weights were measured three times per week with a precision of ±10 mg. The in vivo experiments were conducted in accordance with the Canadian Council on Animal Care (CCAC) guide for care and use of experimental animals. Experimental protocols were approved by the Comité Institutionnel de Protection des Animaux (CIPA) of Université du Québec à Montréal (0621-C1-897-0622).

## 3. Results

**Figure 1:**
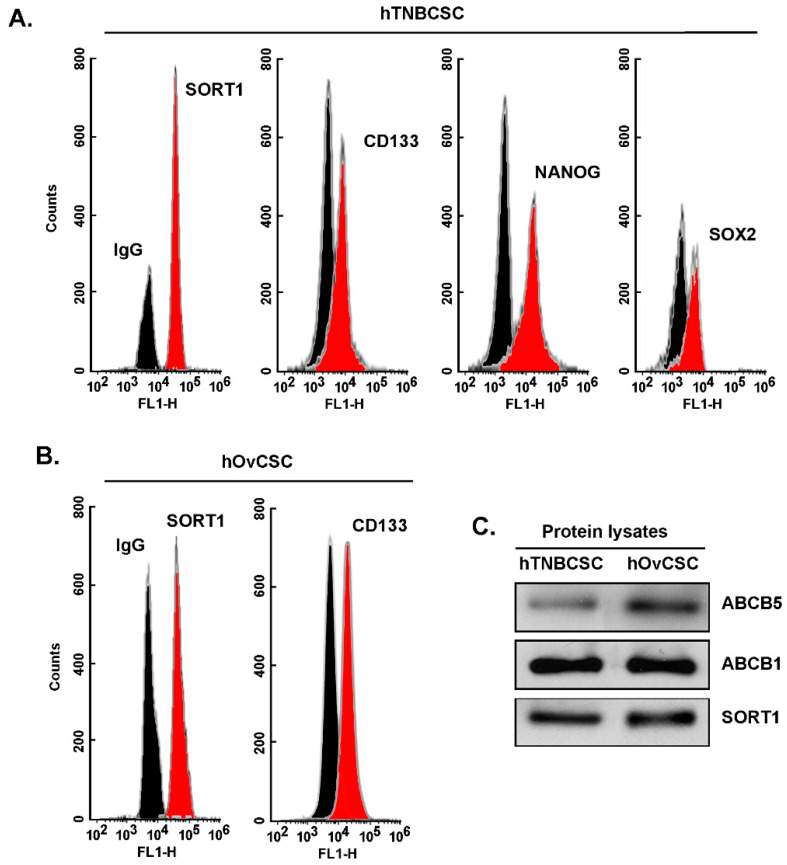
**Immunophenotyping of SORT1 and CSC biomarkers expression in hTNBCSC and hOvCSC.** Immunophenotyping was performed for (**A**) SORT1, CD133, NANOG and SOX2 in hTNBCSC, and for (**B**) SORT1 and CD133 in hOvCSC as described in the Methods section using flow cytometry (IgG is the control Ab isotype, black profile). (**C**) hTNBCSC and hOvCSC cell homogenates (20 µg protein) were separated on a polyacrylamide gel and electrotransferred onto a polyvinylidene difluoride membrane. SORT1, ABCB1, and ABCB5 proteins were detected on the membranes by immunoblotting.

**Figure 2:**
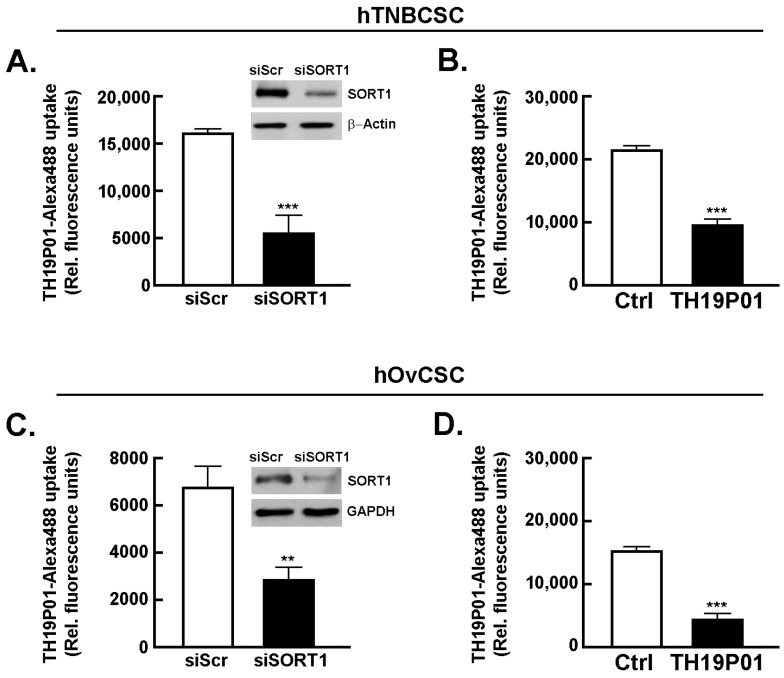
**SORT1 is required for the internalization of TH19P01 in hTNBCSC and hOvCSC.***SORT1* siRNA-mediated transient gene silencing was performed as described in the Methods section, and a Western blot performed to evaluate the extent of SORT1 protein expression decrease in (**A**) hTNBCSC (insert) and (**C**) hOvCSC (insert). siScr is a scrambled random sequence of siRNA and serves as a control. β-Actin or GAPDH were used as a loading controls. Transfected cells were then incubated with fluorescent Alexa^488^-TH19P01 peptide as described in the Methods section. Untransfected cells were also exposed to media supplemented with DMSO alone (Vehicle), or 50 μM TH19P01 to monitor Alexa^488^-TH19P01 peptide uptake for (**B**) hTNBCSC or (**D**) hOvCSC. The fluorescence contained within the cells was measured by flow cytometry after cells were incubated for 2 h, washed and trypsinized. Fluorescence associated with Vehicle alone reflects background fluorescence of the system. The data shown represent means ± SEM, *n* = 3, each performed in duplicate. Statistical comparison was performed using *t*-test, comparing all other samples to the cells incubated with Alexa^488^-TH19P01 alone. Significance was assumed for ** *p* < 0.01. *** denotes *p* < 0.001.

Sortilin is expressed in cancer stem-like cells from TNBC and from ovarian cancer and required for TH1902 intracellular internalization. As assessed by flow cytometry immunophenotyping, commercially available hTNBCSC are expressing CSC markers such as CD133, NANOG, and SOX2 as well as SORT1 (Figure 1A) [https://celprogen.com/ (accessed on 8 August 2020)]. SORT1 and CD133 expressions were also observed in hOvCSC (Figure 1B). SORT1, ABCB1, and ABCB5 protein expression was measured in cell lysates by Western blotting (Figure 1C). Results show that SORT1, as well as ABCB1 and ABCB5, are expressed in the two CSC models. Of interest, stem cells are noted for their expression of ABCB5, a MDR efflux protein and member of the ABC multidrug transporter family [38,39]. Next, the requirement for SORT1 for TH1902 internalization within hTNBCSC and hOvCSC was investigated. SORT1 gene silencing was first achieved through siRNA transient transfection in both cell lines and confirmed using immunoblotting of the respective cell lysates (inserts, Figure 2A,C). Then, uptake of a fluorescently labeled Alexa^488^-TH19P01, the peptide moiety of TH1902, was found reduced in hTNBCSC (Figure 2A) and in hOvCSC (Figure 2C), and significantly blocked by competition due to excess quantities of nonfluorescent TH19P01 (Figure 2B,D) in the respective cell lines. Thus, SORT1 activity, shown by either functional inhibition by excess of the peptide alone or through gene silencing, is required for the internalization of TH19P01 peptide within hTNBCSC and hOvCSC.

**Figure 3:**
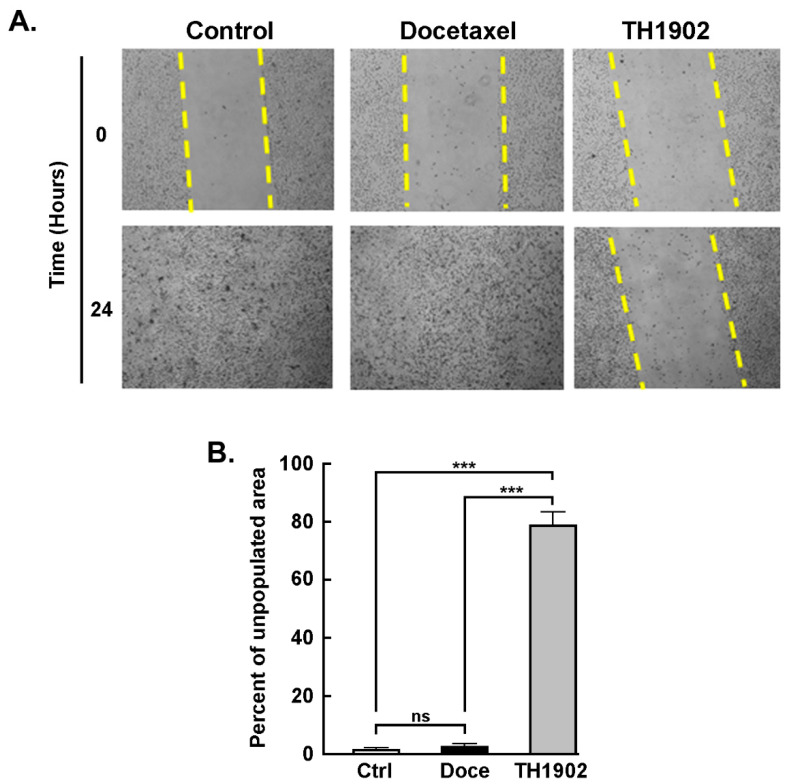
**TH1902 inhibits CSC migration**. (**A**) Pictures of wells bearing confluent layers of hTNBCSC were scratched to denude one area of cells, rinsed with serum-free medium and then treated for 2 h with vehicle (DMSO), 2 µM docetaxel (Doce) or 1 μM TH1902 (equivalent in docetaxel content to the docetaxel treatment) in serum-free media. Cells were then rinsed and incubated in fresh complete media. At 0 and 24 h following the initial wound, cells were washed and then photographed using a phase contrast microscope at a 4× magnification. (**B**) Quantification was performed with ImageJ. Data were obtained from three different experiments and are represented as means +/− SEM (*n* = 3); statistical analysis was performed for each time point using *t*-test (*** signifies *p* < 0.001 for differences between TH1902 and other conditions).

*TH1902 inhibits hTNBCSC migration*. A wound-healing assay was utilized to assess the anti-migratory effects of TH1902 on hTNBCSC. One region of the well bearing confluent cells was scraped clean of cells, and the cells were then incubated for 2 h in the presence of either vehicle (DMSO), 2 µM docetaxel or 1 µM TH1902 as described in the Methods section and pictures taken (Figure 3A). As observed at 24 h following cell removal, the cleared regions which had been subsequently treated with medium containing either vehicle or docetaxel had been completely recolonized due to cell migration. In contrast, the cleared sections which had then been treated with TH1902 remained practically devoid of migrant cells (Figure 3B). This shows that exposure to TH1902 clearly prevented cell migration. Another explanation, given that TH1902 contains two docetaxel molecules, would be that uptake of this molecule would lead to apoptosis or cell cycle arrest, through the subsequent release of docetaxel from TH1902.

**Figure 4:**
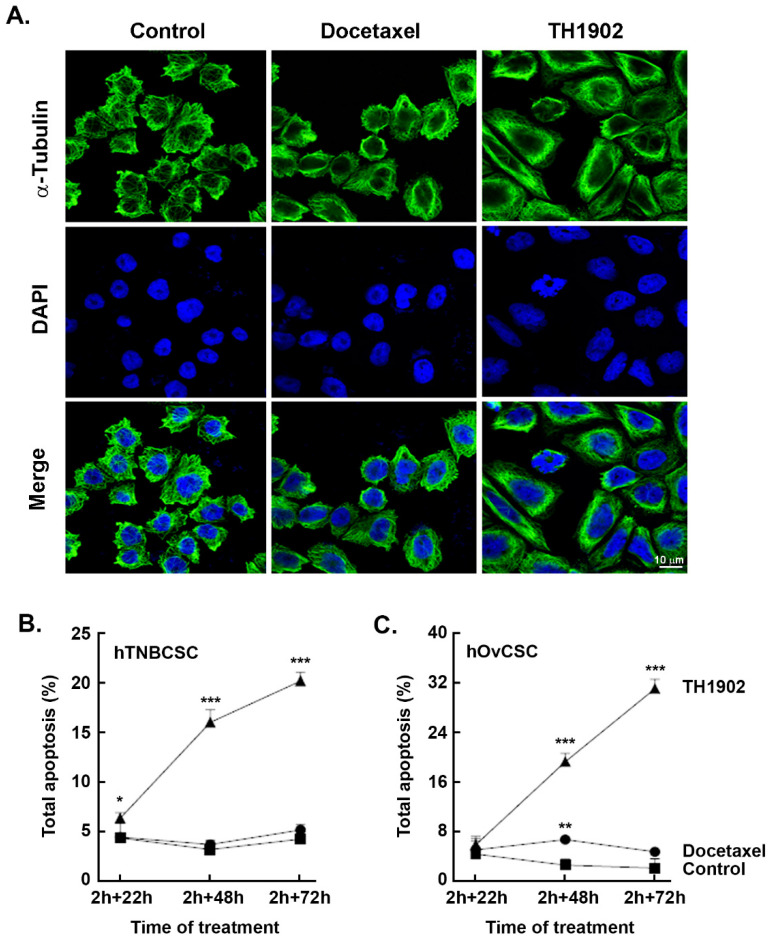

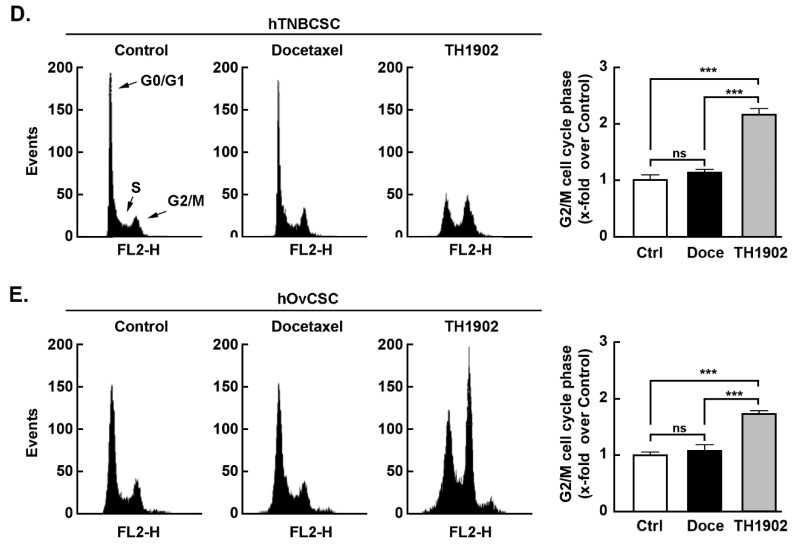
**TH1902 induces morphological changes, apoptosis, and cell cycle arrest in hTNBCSCand hOvCSC.** Cells were treated in medium containing vehicle (DMSO, Control), 4 µM docetaxel or 2 μM TH1902 for 2 h and then incubated in complete medium for 22 to 72 h. (**A**) Treated hTNBCSC were fixed and immunostained with anti-α-tubulin Ab. Cells were then stained with DAPI before imaging by confocal microscopy. Representative pictures from each condition are displayed using fluorescence filters to demonstrate tubulin staining (green) and DAPI staining. The extent of apoptosis in (**B**) hTNBCSC or (**C**) hOvCSC was determined upon similar conditions as described above, and after different intervals of incubation ranging from 22 h to 72 h. Cells were harvested and then flow cytometry performed following staining with annexin V-FITC and propidium iodide (PI). Data were obtained from three different experiments and are represented as means +/− SEM; statistical analysis was performed for each time point using one-way ANOVA with Dunnett’s multiple comparison test (ns = non significant, * signifies *p* < 0.05, ** signifies *p* < 0.01 and *** signifies *p* < 0.001 for differences between TH1902 and control conditions). For cell cycling experiments, (**D**) hTNBCSC or (**E**) hOvCSC were treated for 2 h in medium containing vehicle (DMSO, Control), 4 µM Docetaxel or 2 μM TH1902, then incubated in complete fresh media for 22 h. The DNA content of each cell line after both intervals was analyzed by flow cytometry using FxCycle™ PI/RNase Staining Solution. The experiment was repeated 3–4 times and representative data are shown for hTNBCSC and for hOvCSC. Data are represented as means +/− SEM. Statistical analysis was performed using one-way ANOVA with Dunnett’s multiple comparison test (ns = non significant, *** signifies *p* < 0.001).

*Induction of apoptosis and cell cycle arrest in G2/M by TH1902*. The ability of TH1902 to induce apoptosis in CSC was next examined. hTNBCSC and hOvCSC were exposed to either 2 μM TH1902 or 4 μM docetaxel for 2 h followed by rinsing of the cells and then further incubated in complete medium for up to 72 h. Cells were harvested at each time point and subsequently assessed for apoptosis and cell cycling as described in the Methods section. Morphologically, staining of tubulin with DAPI at 48 h post-treatment led to significant multilobed nuclei and disruption of the microtubules by TH1902 when compared to docetaxel in hTNBCSC (Figure 4A). Furthermore, when cell death was assessed, although partially initiated at 24 h, TH1902 incrementally triggered apoptosis at 48 and at 72 h in both cell lines tested whereas this was absent in docetaxel-treated hTNBCSC (Figure 4B) and hOvCSC (Figure 4C) respectively. Next, as blocking of the depolymerization of microtubules by docetaxel is believed to induce cell cycle arrest [40], CSC cycle progression was further monitored upon TH1902 treatment and compared to that of docetaxel. The distribution of DNA in control cells after 22 h incubation showed that most cells had a single set of chromosomes (G0/G1) while a small proportion (~5%) were in the G2/M phase. Exposure to docetaxel had little impact on this distribution, but exposure to TH1902 produced a large increase in the G2/M population with a commensurate fall in the G0/G1 population. Results were found comparable between hTNBCSC (Figure 4D) and hOvCSC (Figure 4E) and reflect the unique ability of TH1902 to produce cytotoxic effects in both CSC models tested, which are reported to be resistant to established agents such as docetaxel [41,42].

**Figure 5:**
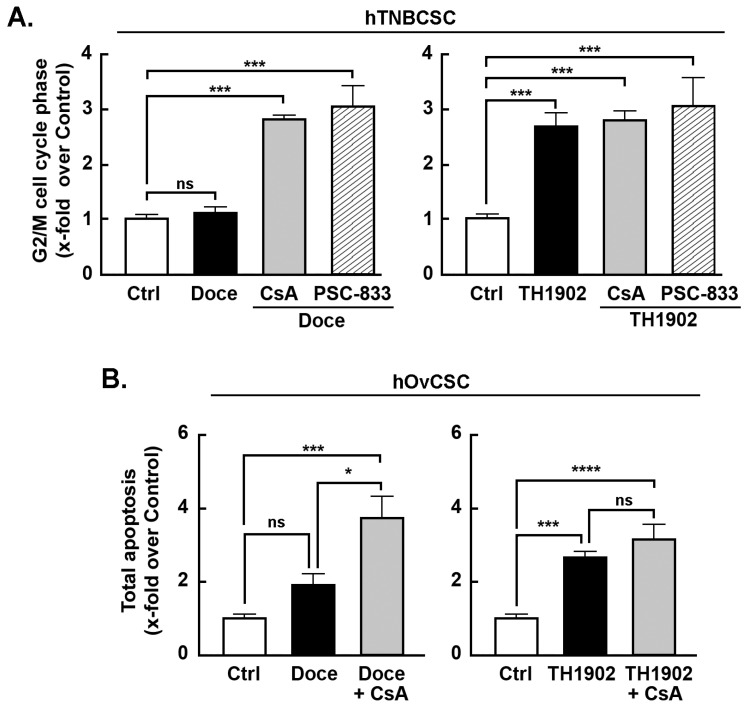
**Effect of MDR inhibitors on cell cycle arrest following treatment with docetaxel or TH1902.** MDR inhibition was performed by pre-incubating hTNBCSC cells for 30 min ± 10 µM CsA (*n* = 4) or PSC-833 (*n* = 3) for cell cycle experiments, or 1 μM for apoptosis assays. Cells were then incubated for 2 h with media containing the same quantities of vehicle, CsA or PSC-833 along with vehicle (Control, DMSO), 4 µM Docetaxel or 2 μM TH1902 for cell cycle experiments, or 1 µM Docetaxel and 0.5 μM TH1902 for apoptosis assays. These media were then replaced by fresh media which continued to have the same levels of CsA or PSC-833. Cells were incubated for 48 h before staining with the FxCycle™ PI/RNase Staining Solution followed by analysis by flow cytometry. Histograms representing the proportion of (**A**) hTNBCSC in G2/M phase are shown. Histograms representing the proportion of (**B**) hOvCSC in apoptosis are also shown. Values shown represent the mean ± SEM from three to four different experiments (each done singly or in duplicate) and statistical analysis was performed using Bonferroni’s multiple comparisons test (ns = non significant, * *p* < 0.05, *** *p* < 0.001, **** *p* < 0.0001). All significant differences are indicated.

*Effects of MDR inhibitors on G2/M cell cycle arrest and apoptosis induced by docetaxel and TH1902*. Multidrug resistance (MDR) proteins are one of the major factors underlying the CSC resistance to chemotherapy of CSC [43]. To better address the impact of the MDR phenotype of hTNBCSC and hOvCSC on both docetaxel and TH1902, inhibitors (CsA and PSC-833) of MDR proteins were tested. When the status of hTNBCSC cells in G2/M phase is compared between docetaxel and TH1902 treatments, the first difference noted is that the number of cells in G2/M phase after 48 h incubation was unaffected by docetaxel (Figure 5A, left panel, black bar) whereas TH1902 caused a strong increase (Figure 5A, right panel, black bar). Docetaxel-mediated cell cycle arrest was, on the other hand, strongly affected by both CsA and PSC-833, two inhibitors of MDR1. In their presence, docetaxel had a pronounced effect that appears identical to that seen with TH1902 (Figure 5A). These results demonstrated that the lack of G2/M phase arrest seen by docetaxel is mainly due to the efflux activity of the MDR proteins found in these cells. In contrast, the cell cycle arrest in G2/M phase induced by TH1902 was unaltered by the presence of these MDR1 inhibitors (Figure 5A) indicating that the cytotoxicity of TH1902 is unaffected by the presence of these MDR proteins. Such effect on cell cycle arrest was, surprisingly, not as significant but with similar tendencies in hOvCSC (not shown) and prompted to investigate whether the apoptotic process was rather sensitive to MDR1 inhibition. Cells were thus treated as described above for hTNBCSC, CsA was found to effectively potentiate docetaxel-induced apoptosis (Figure 5B, left panel, grey bar), whereas it was ineffective to alter TH1902-mediated apoptosis (Figure 5B, right panel, grey bar) suggesting that differential chemoresistance molecular processes can still be effectively circumvented by TH1902.

**Figure 6:**
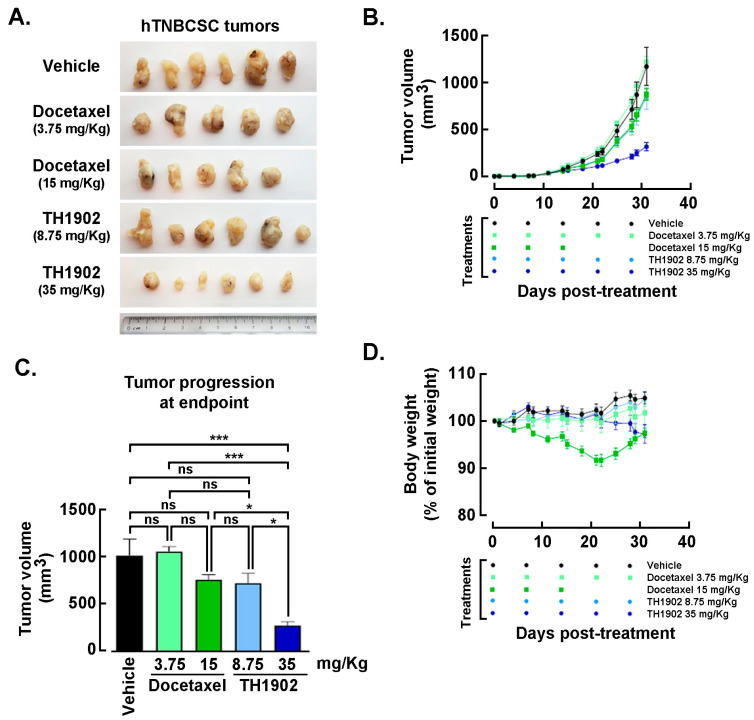

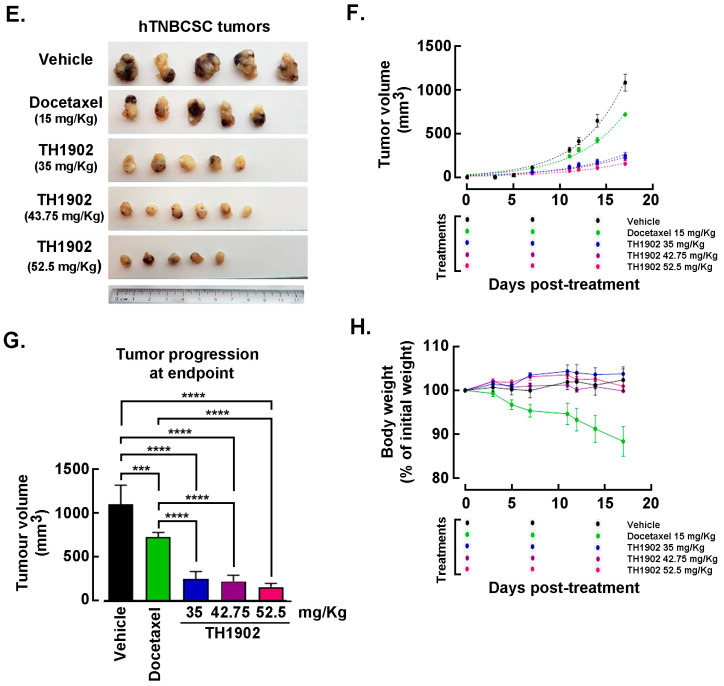
**Growth of hTNBCSC and hOvCSC xenografts within mice following administration of docetaxel or TH1902.** At 3 days following subcutaneous implantation of hTNBCSC or hOvCSC xenografts within immunodeficient mice, animals began receiving weekly administration of vehicle, docetaxel (3.75 or 15 mg/kg) or TH1902 (8.75, 35, 42.75 or 52.5 mg/kg, first two containing docetaxel levels equivalent to those in the two docetaxel dosages). The tumor pictures were taken for (**A**) hTNBCSC and (**E**) hOvCSC, and size (**B**,**F**) was measured manually at regular intervals in order to monitor the effects of test article administration on tumor growth. Symbols represent means ± SEM, *n* = 5 to 6. Note that there is virtual overlap between the 15 mg/kg docetaxel curve and the 8.75 mg/kg TH1902 curve. The symbols displayed below the abscissa denote the days on which treatments occurred. Note also that docetaxel (15 mg/kg) was only administered three times as that combined quantity matches the maximum tolerated dose (MTD) for docetaxel in mice. Statistical comparison between the different treatments of (**C**) hTNBCSC or (**G**) hOvCSC was performed by calculating the change in tumor sizes between the first day of treatment and the last day of the experiment. The sizes were compared by one-way ANOVA and the mean values for tumor sizes associated with each treatment were compared to the tumor sizes for vehicle-treated animals using Dunnett’s multiple comparison test with *p* < 0.05 as the pre-set level of significance (ns = non significant, * *p* < 0.05, *** *p* < 0.001, **** *p* < 0.0001). During the treatments, body weights of the mice were routinely recorded and considered as a rough estimate of morbidity. The data is shown here as a percentage of the animal’s body weight at the beginning of treatment (**D**,**H**). Symbols represent means ± SEM, *n* = 5 to 6. The symbols displayed below the abscissa indicate the days on which treatments occurred. The treatments listed along the abscissa identify the treatment along with the concentration used (in mg/kg) listed in brackets.

*Effects of Docetaxel and TH1902 administration on in vivo growth of hTNBCSC and hOvCSC xenografts.* In vivo assessment in tumor-bearing animal models classically complements cell cultures data for comparing cancer treatment efficacies. For this reason, hTNBCSC and hOvCSC xenografts were implanted subcutaneously into immunodeficient mice as described in the Methods section with only 1000 cells given their highly tumorigenic nature. Three days later, the animals began receiving weekly IV bolus administration of either vehicle, docetaxel, or TH1902. Docetaxel was administered at a dose (15 mg/kg/week; for 3 cycles) in accordance with the estimated maximal tolerated dose (MTD) for mice [44], as well as at ¼ of the MTD (3.75 mg/kg/week). TH1902 was administered at doses (35 and 8.75 mg/kg/week) which contained quantities of docetaxel equivalent to those in the two administrations of free docetaxel. From the size of the hTNBCSC (Figure 6A) and hOvCSC (Figure 6E) tumors, it is apparent that docetaxel had little impact on xenografts growth when administered neither at its MTD, for both hTNBCSC (Figure 6B) and hOvCSC (Figure 6F), nor at ¼ of this dosage as reflected by the growth curves. TH1902, when administered at a dosage equivalent to docetaxel at its MTD, provided greater tumor growth inhibition than did docetaxel for both xenograft models. Furthermore, higher dosage of administered TH1902 (up to 1.5 equivalent of docetaxel MTD) did not generate significant differences in terms of hOvCSC tumor inhibition without affecting mice body weights suggesting that TH1902, even at higher doses, is better tolerated compared to docetaxel (Figure 6B). Then, in order to statistically compare the effects of docetaxel and TH1902 on tumor growth, the tumor sizes measured at the vehicle group endpoint were compared and statistically significant differences between the tumor sizes in vehicle-treated animals found in TH1902-treated animals for hTNBCSC (Figure 6C) and hOvCSC (Figure 6G). Mouse body weight was used as an indicator of the morbidity associated with administration of docetaxel or TH1902. Administration of docetaxel at its MTD provoked a weight loss that approached 10% after the treatments, which is often observed in xenograft models with administration of docetaxel at this level. The body weights of animals treated with an equivalent quantity or a 1.5-fold equivalent of TH1902 were maintained at a roughly constant level throughout the experiment. The animals treated with vehicle recorded a slight weight gain (~5%) over this period while animals treated with the lower dosages of docetaxel or TH1902 were similar to the animals treated with vehicle (Figure 6D,H). The body weight data indicates that TH1902 appears to be better tolerated than the equivalent quantity of free docetaxel, in addition to the fact that TH1902 is more efficacious than docetaxel when administered in vivo in the murine models of CSC tested.

**Figure 7:**
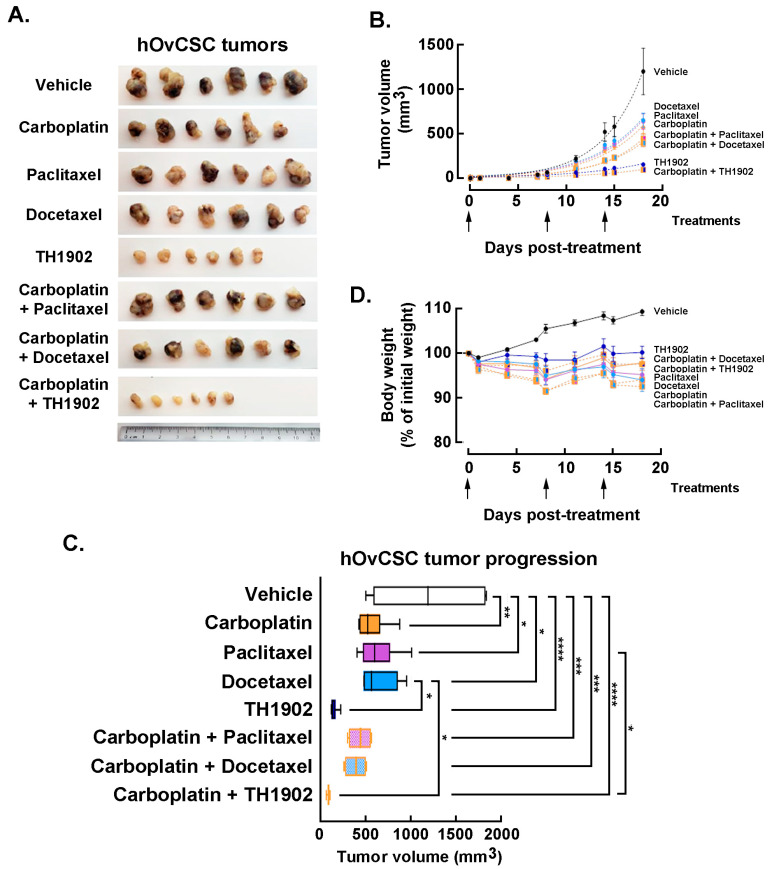
**Growth of hOvCSC xenografts within mice following single agents or combination administration of TH1902, paclitaxel, or docetaxel to TH1902**. (**A**) Mice bearing hOvCSC xenografts were treated with either single articles (40 mg/kg/week carboplatin, 10 mg/kg/week paclitaxel, 10 mg/kg/week docetaxel or 23 mg/kg/week TH1902) or with the single taxanes or TH1902 combined with carboplatin. Following euthanasia, tumors were excised from the mice, fixed and then photographed. The ruler shown at the bottom for scale is numbered in centimeters. (**B**) Groups of mice bearing hOvCSC xenografts were administered IV Vehicle, docetaxel, TH1902 or paclitaxel with and without IP injection of carboplatin. The arrows shown beneath the abscissa indicate the days at which all treatments were administered. Symbols represent means ± SEM, *n* = 6 for all groups. The dotted lines joining each set of points are exponential curves calculated to best fit each group. (**C**) Tumor sizes on day 18 (final day of experiment) were measured for all animals and these sizes were compared between the groups receiving different treatments. The box ends represent the median values of the lower and upper halves of the datasets and the whiskers show the minimum and maximum values of each set. The vertical line inside the box marks the median value for that set. Pairwise comparisons that are not marked in the figure were not statistically significant. Statistical comparison was performed using a one-way ANOVA followed by Tukey’s multiple comparisons test with statistical significance pre-set at *p* < 0.05. * denotes *p* < 0.05, ** denotes *p* < 0.01, *** denotes *p* < 0.001 and **** denotes *p* < 0.0001. *n* = 6 for all groups. (**D**) Groups of mice bearing hOvCSC xenografts were treated with either vehicle or with carboplatin, docetaxel, TH1902 or paclitaxel, or with combinations of carboplatin along with the other three articles. Only minor weight changes were associated with any of the treatment regimens. The arrows shown beneath the abscissa indicate the days on which treatments occurred. Symbols on the graph represent means ± SEM, *n* = 6 for all groups. The lines connecting the points have no additional significance other than those dotted lines connect the points representing combined administration of carboplatin along with a taxane or TH1902.

*In vivo impact of carboplatin administration combinations to TH1902, paclitaxel, or docetaxel.* Mice bearing hOvCSC xenografts were either singly administered vehicle, docetaxel, paclitaxel, TH1902 or carboplatin; other groups of mice were administered carboplatin along with either docetaxel, paclitaxel or TH1902. Due to the unknown effects of combining these treatments, the dosages used for each article (both administered singly or in combination) were reduced to the following: docetaxel 10 mg/kg; TH1902 23 mg/kg/week (equivalent to the docetaxel dosage); paclitaxel 10 mg/kg; carboplatin 40 mg/kg, and tumor volumes measured as described in the Methods section. The inhibition of tumor growth by docetaxel was again obviously inferior to that of TH1902 (Figure 7A). Paclitaxel and carboplatin each exhibited a level of tumor growth inhibition practically equivalent to that seen with docetaxel. Administration of either taxane combined with carboplatin administration produced a further small decrease in tumor growth. The inhibitory effect on tumor growth by TH1902 was much greater than that of either taxane (with or without carboplatin) and it was slightly increased by combined administration with carboplatin (Figure 7B). The extent of tumor growth inhibition by TH1902 was sufficiently great that it became difficult to demonstrate increased tumor growth inhibition by combining administration of the PDC along with carboplatin. However, the two articles clearly showed enhanced ability to inhibit tumor growth when combined.

Statistical comparison of the inhibition of tumor growth was next performed using the final tumor sizes measured on day 18 following the beginning of treatment (Figure 7C). Carboplatin, paclitaxel and docetaxel (administered individually) all provided significant inhibition of tumor growth compared to the growth measured in vehicle-treated animals; the effect of any of these articles was not significantly different in magnitude from those of the other two articles. Tumor growth in TH1902-treated animals was significantly lower than that measured in vehicle-treated or docetaxel-treated animals. When articles were administered along with carboplatin, the combinations including either taxane or TH1902 produced significant inhibition of tumor growth. In addition, TH1902 plus carboplatin resulted in tumor sizes that were significantly smaller than those seen in animals receiving either of the taxanes as single articles.

For greater sensitivity in detecting a difference between single groups, the effect of combining other articles with carboplatin was also assessed by nonlinear regression of each group’s tumor sizes over the length of the experiment and comparing two or more sets of data to see if it could be fit with a single curve or required separate curves. For docetaxel, paclitaxel and TH1902, the addition of carboplatin required a separate curve fit, indicating a significant change (*p* < 0.0001) in the tumor growth (not shown). The combination of TH1902 with carboplatin produced a small but significant increase in the inhibition of tumor growth compared to TH1902 alone; this effect could only be of a limited extent since the effect of TH1902 alone inhibited most of the tumor growth. The extent of tumor growth inhibition by TH1902 was sufficiently great that it became difficult to demonstrate increased tumor growth inhibition by combining administration of the PDC along with carboplatin. In conclusion, as was seen with tumors excised following treatment with single agents, the tumors excised following treatment with combined agents showed much smaller tumors following treatment with TH1902 (either alone or combined with carboplatin) (Figure 7A). Although the treatments with combined articles produced tumors that measured smaller than those with single articles did, the difference is not visually apparent. The superiority of TH1902 to taxanes and carboplatin for inhibiting tumor growth of these cancer stem cell-like xenografts is an evident conclusion.

Finally, the effects of combined treatment regimens on body weight was assessed. A slow increase in body weight was observed for the animals receiving vehicle, whereas the body weights of the mice administered any of the three combination treatments remained fairly constant, similar to those of the mice receiving single articles (Figure 7D). This indicates an absence of serious side effects associated with co-administration of TH1902 with carboplatin or with the other co-administrations. The absence of strong weight loss associated with docetaxel administration is presumably due to the docetaxel dosage being reduced in this experiment (2/3 of docetaxel MTD dose). Altogether, these observations suggest that TH1902 therapeutic window appears efficient as either a single agent treatment or in combination with carboplatin.

## 4. Discussion

The recurrence of cancers treated with current chemotherapeutic agents, in part attributable to the survival of the CSC subpopulation as well as the possible dedifferentiation of tumor cells into stem cells, indicates that one of the optimum approaches to tumor eradication would be the combined targeting of both the differentiated tumor cells and the CSC [45]. Increasing evidence suggests that the accumulation of therapy resistant CSC population within TNBC contributes to poor clinical outcomes. These CSC are enriched in TNBC compared to non-TNBC breast cancers [46]. It has been demonstrated that about ~2% of the TNBC-derived MDA-MB-231 tumor cells are believed to be CSC [35,47]. The initially reported successful elimination of MDA-MB-231 tumor cells by TH1902 confirms the efficacy of this treatment against tumor cells and suggests that it may have been also active against CSC. In addition, the present demonstration that SORT1 is expressed in the hTNBCSC and hOvCSC models tested, along with the functional evidence that SORT1 activity is required for incorporation of the peptide backbone of TH1902 into CSC, provides a logical framework for interpreting the results presented here on these models. It is also the first demonstration of SORT1 protein expression in cancer stem-like cell models that provides support to the SORT1 activity involved in the propagation of stem cells in several types of cancers [48].

The effects of TH1902 on both the overall cancer cell compartment and the more specific CSC niche positions this PDC among the promising therapeutic strategies to impact the development of human SORT1+ cancers and to reduce the risk of recurrence. The additional therapeutic advantage of TH1902 is to circumvent the MDR1 transporters functions, leading to cell cycle arrest in G2/M, and to increased induction of apoptosis in CSC. Previous work examining the effects of TH1902 on TNBC-derived MDA-MB-231 xenograft growth displayed complete and lasting tumor regression [24]. One reason that this was surprising was that this cell line contains up to 2% of CSC, which are well-known as being difficult to eliminate by chemotherapy or radiotherapy [49,50]. TH1902 also completely inhibited in vitro vasculogenic mimicry (VM) by ES2 ovarian cancer and MDA-MB-231 TNBC cells at low concentrations, another process responsible for chemotherapy resistance and cancer recurrence, and which is often associated with an active CSC population [27]. The simultaneous pro-apoptotic and cell cycling trapping activities of TH1902 against CSC may provide the first molecular evidence supporting a SORT1-mediated inhibition of VM of these cells once docetaxel is released intracellularly from the PDC. Such combined cellular effects of docetaxel have been reported previously in renal clear cell carcinoma [51]. In addition, it has been reported that CD133+ and ABCB5+ subpopulations colocalized in melanomas in perivascular niches that contain vascular endothelial (VE)- cadherin+ melanoma cells, which can form VM [52]. Interestingly, the anti-VM process observed at pM TH1902 concentrations, contrasted with the ability of docetaxel to only inhibit VM at much higher concentrations as documented previously [26].

In vivo assessment of docetaxel and TH1902 using a murine CSC xenograft system showed that weekly IV administration of docetaxel had little impact on tumor growth, whereas a similar administration of TH1902 provided significant inhibition of the CSC xenografts growth. In addition, considerable weight loss was observed when mice were treated with docetaxel at its MTD, whereas the equivalent or 1.5-fold higher quantity of TH1902 had non-significant effect on body weight, suggesting that the PDC was better tolerated. Thus, TH1902 appears to be superior to docetaxel for the treatment for SORT1-expressing tumors partly due to its effect against CSC, which in turn appears to be the result of the unique means by which this treatment enters the cells and bypasses MDR proteins at their cell surface. As expected, mice treated at the MTD for docetaxel were losing significant weight and seemed to start recovering a week after the treatments stopped. In contrast, body weight of mice treated with higher doses of TH1902 was unaffected when compared to body weight of mice treated with the vehicle. This indicates that it is less of a strain for the body to accept this treatment rather than docetaxel.

The activity of paclitaxel in advanced ovarian cancer, both as a single agent and in combination chemotherapy, has been demonstrated in numerous phase I/II trials [53]. Paclitaxel/platinum combinations have produced encouraging results in phase III trials and are now considered standard therapy for advanced disease. Carboplatin, an effective but more tolerable analog of cisplatin, has been substituted for cisplatin to reduce the toxicity of the paclitaxel/cisplatin regimen [54]. In a preliminary analysis, paclitaxel/carboplatin appeared to be better tolerated with similar efficacy from paclitaxel/cisplatin. Here, the documented improved toxicity profile and capacity to circumvent the CSC chemoresistance phenotype to docetaxel as either a single agent or in combination modalities with carboplatin further demonstrates that such combination has a higher therapeutic index. Overall, these are the first results indicating that TH1902, alone or in combination with carboplatin, may be preferred over taxanes/carboplatin combinations in the targeting of the ovarian cancer stem-like cell phenotype for advanced ovarian cancer.

## 5. Conclusions

Our study reports the first comprehensive evidence indicating the potential therapeutic impact of TH1902 would offer against the CSC sub-population known to reside within ovarian cancer and TNBC. Given the current molecular demonstration that exploiting SORT1 function to internalize TH1902 within these CSC further enables to circumvent their chemoresistance phenotype provides the rationale that such therapy may also impact on the tumor microenvironment of the CSC niche. Finally, the future of anti-CSC therapy with TH1902 appears promising, both in experimental settings and in clinical trials, against cancer cells showing plasticity, metastatic potential, and resistance against anti-cancer treatment.

## Data Availability

The data generated and/or analyzed during the current study are not publicly available due to pending patent application, but will be made available from the corresponding author on reasonable request.

## Abbreviations

ABCB1, ATP binding cassette subfamily B member 1; ABCB5, ATP binding cassette subfamily B member 5; CSC, Cancer stem cells; IP, Intraperitoneal; IV, Intravenous; MDR, Multi-drug resistance; MTD, Maximal tolerated dose; PDC, peptide-drug conjugate; PI, Propidium iodide; P-gp, Permeability-glycoprotein; SORT1, Sortilin; TNBC, Triple-negative breast cancer; VM, vasculogenic mimicry; hTNBCSC, Human triple-negative breast cancer stem cells; hOvCSC, Human ovarian cancer stem cells.
